# Vascular Protective Effects of* Morinda citrifolia* Leaf Extract on Postmenopausal Rats Fed with Thermoxidized Palm Oil Diet: Evidence at Microscopic Level

**DOI:** 10.1155/2018/6317434

**Published:** 2018-09-05

**Authors:** C. L. G. Chong, Faizah Othman, Farida Hussan

**Affiliations:** ^1^Department of Anatomy, Faculty of Medicine, Pusat Perubatan Universiti Kebangsaan Malaysia, Jalan Yaacob Latif, Bandar Tun Razak, 56000 Cheras, Kuala Lumpur, Malaysia; ^2^Human Biology Division, School of Medicine, International Medical University, 126 Jalan Jalil Perkasa 19, 57000 Bukit Jalil, Kuala Lumpur, Malaysia

## Abstract

Atherosclerosis is now well understood as an inflammatory disease instead of lipid storage disorder; however, the conventional treatment is not targeted on treating the inflammation.* Morinda citrifolia* L. (Rubiaceae) leaf or noni leaf, which is a medicinal food (*ulam*) used in Traditional Malay Medicine to prevent chronic diseases, may have the potential to be formulated into a functional antiatherosclerotic agent. This study aimed to investigate the effectiveness of* Morinda citrifolia* leaf extract (MCLE) treatment at histological and ultrastructural level, comparing it with Simvastatin. Thirty-eight female Sprague Dawley rats were divided into five groups: Sham (Sham), ovariectomized (OVX), ovariectomized with Simvastatin 10 mg/kg (OVX+ST), ovariectomized with low dose MC 500 mg/kg (OVX+MCLD), and ovariectomized with high dose MC 1000 mg/kg (OVX+MCHD). Atherosclerosis was induced by producing oestrogen deficiency through ovariectomy and feeding with thermoxidized palm oil (TPO) diet for 12 weeks along with the treatment. The results revealed significantly (P<0.05) lower systolic blood pressure (SBP) in the group treated with MCHD compared to the untreated OVX, whereas the diastolic blood pressure (DBP) was significantly higher in the untreated OVX group compared to the Sham group. Treatment with MCHD also significantly lowered the total cholesterol (TC) level compared to the OVX. The OVX group showed significantly lower high-density lipoprotein (HDL) level compared to the Sham group. The untreated OVX group showed evident histological and ultrastructural features of vascular inflammation such as blood cells accumulation in the lumen, vacuolation of the endothelial cells, subendothelial space widening, elastic fibres disruption, increased intima media thickness (IMT), smooth muscle cells fragmentation, and perivascular adipose tissue (PVAT) deposition. All these pathological changes were less seen in the groups treated with MCLE. In conclusion, we reported the mechanism of antiatherosclerotic property of MCLE through lipids elimination and anti-inflammatory activity. In addition, we do not recommend the use of statin in the absence of dyslipidemia as it causes PVAT deposition.

## 1. Introduction

Cardiovascular disease (CVD) remains one of the major causes of morbidity and mortality worldwide [1] and it is manifested by atherosclerosis. Atherosclerosis is defined as a chronic disorder of large and medium sized arterial wall [2] characterized by endothelial dysfunction, vascular inflammation, and lipid deposition in the intima [3]. These changes are followed by plaque deposition, vascular remodeling, luminal stenosis, and tissue ischemia [4]. The methods of treatment and prevention involved lifestyle modification such as exercise and well-balanced diet; however, it is difficult to achieve or maintain in patients. Statins are widely prescribed for the treatment and primary prevention of CVD but the use of the drugs are limited by the presence of unpleasant side effects such as myopathy [5]. Thus, novel therapeutic strategies and highly effective alternative treatment are needed to overcome the impact of this disease.

The use of thermally oxidized oil or thermoxidized palm oil (TPO) in cooking is common especially in fried and processed foods [6]. The cooking oil is reused repeatedly in order to save costs. The consumption of such oil is hazardous to health because exposure to high temperature during the frying process decreases the antioxidant content of the oil, increases lipid peroxidation, and generates free radicals-induced oxidative stress [7]. Previous studies have shown that chronic ingestion of food prepared by reheated oil leads to hypertension [8] and atherosclerotic changes in the blood vessel [9]. These detrimental effects are more pronounced in postmenopausal women due to loss of protective effects of oestrogen [10]. In vivo study also showed that postmenopausal rats fed with TPO diet developed atherosclerotic lesion in the aorta [11].

*Morinda citrifolia* (MC) leaf used in traditional medicine dates back thousands of year [12]. MC leaf is consumed raw as vegetable salad called “*ulam*” and “*kerabu*” in Traditional Malay Medicine to prevent hypertension and aging and to invigorate the blood [13]. Phytoactive substances isolated from the leaf include *β*-carotene [14], kaempferol [15], quercetin, rutin [16], and ursolic acid [17]. These substances were shown to possess anti-inflammatory [18, 19], antidyslipidemic [20], and antioxidant activities [21]. This study aimed to validate the traditional uses of MC leaf extract (MCLE) as a medicinal food in Traditional Malay Medicine [22] by investigating the protective effects of the leaf extract on the aortic structure and cardiovascular function in ovariectomized rats fed with TPO diet for a period of 12 weeks, with emphasis on the aortic tissue histological and ultrastructural findings. To the best of our knowledge, there is no other study done on the effect of MCLE on the aortic ultrastructure.

## 2. Material and Methods

### 2.1. Preparation of* Morinda citrifolia* Leaf Extract

The powdered form of MC ethanolic leaf extract was obtained from Prof. Dr. Suhaila Mohamed from Institute of Bioscience, Universiti Putra Malaysia. The extract was prepared by the following procedures: The fresh young leaves of MC were collected from Bukit Expo, Universiti Putra Malaysia, and authenticated by an expert botanist. Voucher specimen was available and deposited at the herbarium of the university department. The fresh leaves were washed, ground, and homogenized with water. Then, equal volume of 70% ethanol was added, soaked for three hours, and filtered. The filtrate was put into a rotary evaporator after which the resultant green paste was added with 20% starch to form powder. It was then dried in the oven and packed in polythene bags with nitrogen purge. The extract was administered at the dosage of 500 mg/kg/day and 1000 mg/kg/day to the respective treatment groups via oral gavage for 12 weeks [20].

### 2.2. Preparation of Thermoxidized Palm Oil Diet

The thermoxidized palm oil (TPO) diet was formulated daily in our laboratory by adding five-time heated palm oil (15% w/w) into standard rat chow [9]. Fresh palm oil without cholesterol (Lam Soon Edible Oil, Malaysia) was reheated for five times through deep frying process as described by Owu et al., 1998 [23]. Briefly, 2.5 litres of fresh palm oil was used to fry 1 kg of sweet potatoes in a stainless steel deep fryer at 180°C for 10 minutes. Then, the heated palm oil was cooled down to room temperature for five hours after which it was reused to fry the next batch of sweet potatoes. The entire frying process was repeated for four times to produce five-time heated palm oil. No fresh oil was added to replace the volume loss during the frying process. The test diet was fed to the ovariectomized rats for 12 weeks along with the treatment.

### 2.3. Experimental Animals

Thirty-eight healthy female Sprague Dawley rats (n=38), 3-6 months old, weighing 250-300g, were obtained from the Laboratory Animal Resource Unit, Universiti Kebangsaan Malaysia. The rats were housed individually in a plastic cage with room temperature of 27±2°C, 12 hours light/dark cycle, adequate ventilation, and* ad libitum* access to food (rat chow from Gold Coin, Selangor, Malaysia) and water. All the animal handling procedures were approved by the Animal Ethical Committee of Universiti Kebangsaan Malaysia (UKMAEC approval number FP/ANAT/2014/KHIN/24-SEPT./610-SEPT.-2014-JUNE-2016).

### 2.4. Study Design

The rats were randomly divided into five groups. One group was Sham operated to simulate surgical stress and the other four groups were ovariectomized to produce postmenopausal oestrogen-deficient state. Group 1 (Sham, n=7) underwent mock surgery and was fed with standard rat chow. Group 2 (OVX, n=7) was ovariectomized and fed with TPO diet. Group 3 (OVX+ST, n=8) was ovariectomized, fed with TPO diet, and treated with oral Simvastatin 10 mg/kg/day [24]. Group 4 (OVX+MCLD, n=8) was ovariectomized, fed with TPO diet, and supplemented with low dose MC 500 mg/kg/day. Group 5 (OVX+MCHD, n=8) was ovariectomized, fed with TPO diet, and supplemented with high dose MC 1000 mg/kg/day. Bilateral ovariectomy was performed under general anaesthesia after 1 week of acclimatization period. Briefly, a midline skin incision was made on the abdomen and the periovarian fat pad was identified together with the ovary and the oviduct. The ovary was exteriorized and cut. The same approach was repeated to remove the contralateral ovary. The incision was closed with 4/0 absorbable catgut suture and 4/0 nonabsorbable silk suture (Merck, Germany). Postoperative antibiotic enrofloxacin (Baytril, Korea) was given intramuscularly for seven days. Blood pressure measurement was done after three months of treatment. At the end of the 12 weeks of treatment period, the rats were fasted overnight and sacrificed with diethyl ether (Sigma, Germany). Blood and aortic tissues were collected. The success of ovariectomy was confirmed at necropsy by observation of marked atrophy of the uterine horns.

### 2.5. Blood Pressure Measurement

Blood pressure was measured with noninvasive tail cuff method by using CODA Noninvasive Blood Pressure Acquisition System (Kent Scientific Corporation, USA). Five valid readings of systolic blood pressure (SBP), diastolic blood pressure (DBP), and mean arterial pressure (MAP) were obtained from each rat and the average readings were calculated.

### 2.6. Serum Biochemical Analyses

The whole blood samples were collected via cardiac puncture, placed into plain tube, and immediately sent to PathLab & Clinical Laboratory Sdn. Bhd., Malaysia, for serum analyses of lipid profile and TC/HDL.

### 2.7. Histological Analyses and Histomorphometry

The thoracic aorta tissues were dissected immediately after sacrificing the rats and fixed in 10% formalin for 1 week with change of formalin solution to remove traces of blood. The samples were then dehydrated and embedded in paraffin wax. Thin sections (5 *μ*m) of the aortic tissues were cut and stained with haematoxylin and eosin (H&E) for light microscopy. Aortic sections were also stained with Verhoeff Van Gieson (VVG) stain to identify the elastic fibres. For histomorphometry, four images of the stained samples were obtained at 0°, 90°, 180°, and 270° by using image analyser software (AxioVision LE). The average thickness of tunica intima (TI), tunica media (TM), and intima media thickness (IMT) were obtained. The average thickness values of TI and TM were calculated by the following formula: average thickness of TI and TM (*μ*m) = (measurements at 0°, 90°, 180°, 270°)/4, IMT (*μ*m) = TI + TM [25].

For qualitative electron microscopy study, 0.5 mm thick aortic tissues were obtained from two rats from each group. The tissues were rinsed with 0.1 M phosphate buffer saline (PBS) and fixed with glutaraldehyde fixative. Then, the aortic tissues were washed three times with 0.1 M PBS, treated with 1% osmium tetroxide for 1-2 hour, and washed in distilled water for three times. Next, the tissues were stained with uranyl acetate, dehydrated in ascending series of ethanol solution, and infiltrated in propylene oxide followed by resin for 24 hours. The tissues were embedded in resin at 70°C. Semithin sections were collected and stained with toluidine blue, and areas of interest were identified under light microscopy. Ultrathin sections of the areas of interest were obtained using a diamond knife and were stained with 30% uranyl acetate and Reynolds' lead citrate. The results were viewed by two expert observers under transmission electron microscope (TEM) Tecnai G2 model in a double blinded fashion.

### 2.8. Statistical Analysis

All data were presented as mean ± standard error mean (SEM). Statistical significance level was set at P<0.05 with Confidence Interval of 95%. Normally distributed data were analysed by parametric test using Analysis of Variance (ANOVA) followed by post hoc Tukey. The statistical analysis was performed by using Statistical Package for Social Sciences (SPSS) software version 22 (SPSS Inc., Chicago, USA).

## 3. Results

### 3.1. Observation of the Animals

After two weeks of the ovariectomy, all the rats developed significant weight gain compared to the Sham group. Behavioral changes such as hyperphagia, decreased ambulation, and decreased activity were observed. However, no mortality was observed throughout the experimental period.

### 3.2. Blood Pressure Measurement

Treatment with high dose MC (OVX+MCHD) significantly lowered (P<0.05) the SBP (106.13±3.54 mmHg) compared to the untreated OVX group (120.00±1.62 mmHg). The untreated OVX group showed significantly higher DBP (90.43±2.33 mmHg) compared to the Sham group (72.43±2.71 mmHg). The MAP was significantly lowered in OVX+MCLD (84.25±2.74 mmHg) and OVX+MCHD (79.50±3.88 mmHg) compared to the OVX group (100.57±1.76 mmHg), whereas the OVX group showed significantly higher MAP compared to the Sham group (80.29±3.84 mmHg). The data was summarized in Table 1.

### 3.3. Serum Lipid Profile

Feeding of TPO diet in ovariectomized rats did not cause significant elevation (P>0.05) in the serum total cholesterol (TC), triglycerides (TG), and low-density lipoprotein (LDL) levels. However, treatment with high dose MC (OVX+MCHD) significantly decreased (P<0.05) the TC level (0.49±0.10 mmol/L) compared to the untreated OVX group (0.78±0.06 mmol/L). The untreated OVX group showed significantly lower HDL level (0.43±0.04 mmol/L) compared to the Sham group (0.67±0.05 mmol/L). The TC/HDL was significantly higher in the OVX+MCLD (4.25 ± 0.40) and the OVX group (4.10 ± 0.16) as compared to the normal Sham group (3.11 ± 0.12). The data was summarized in Table 1.

### 3.4. Histomorphometry of the Aortic Wall

No significant difference (P>0.05) was observed in the TI thickness in all the groups. The group treated with high dose MC (OVX+MCHD) showed significantly decreased TM thickness (153.06±3.28 *μ*m) compared to the untreated OVX group (168.24±5.79 *μ*m). The untreated OVX group showed significant thickening of the TM (168.24±24±5.79 *μ*m) compared to the normal Sham group (150.70±2.81 *μ*m). Similarly, the OVX+MCHD showed significantly decreased IMT (161.48±3.90 *μ*m) compared to the untreated OVX (178.56±6.56 *μ*m), whereas the OVX group showed significantly increased IMT (178.56±6.56 *μ*m) compared to the Sham group (158.89±3.2 *μ*m). The data was summarized in Table 2.

### 3.5. Histological and Ultrastructural Assessment

**Figure 1:**
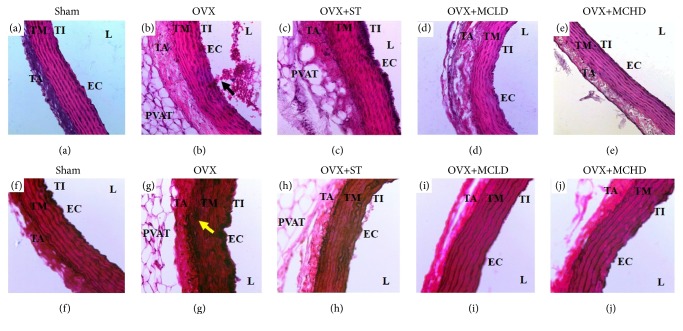
Photomicrograph showing the effect of MC leaf extract on the aorta. (a-e) H&E staining of the rat aortae (X200). Note the presence of infiltratory cells along the aortic wall (arrow) and deposition of perivascular adipose tissue (PVAT) in the tunica adventitia of (b) OVX. (f-j) VVG staining of the rat aortae (X200) showing total disruption of the internal elastic laminae with thickening of the tunica media in (g) OVX. TI: tunica intima, TM: tunica media, TA: tunica adventitia, L: lumen, EC: endothelial cell.

Normal histological features of the aorta were observed under light microscopy in the Sham, OVX+MCLD, and OVX+MCHD groups where distinct histological layers were observed (Figures 1(a), 1(d), and 1(e)). Generally under the light microscope the TI is not easily observed except for the darkly stained nuclei of the endothelial cells (EC) which appeared bulging along the luminal circumference of the vessel. These EC appeared flattened and lie adjacent to the internal elastic laminae (IEL) giving the appearance of a normal TI. The OVX+ST showed thickening of the EC evidenced by the darkly stained nuclei layer seen along the luminal surface of the vessel (Figure 1(c)). In the untreated OVX group (Figure 1(b)), thickening of the TM was noted. The TA was disrupted and infiltrated with perivascular adipose tissue (PVAT) in the OVX, OVX+ST, and OVX+MCLD groups. However, the PVAT seen in the OVX+MCLD was minimal. The lumen of the untreated OVX group showed massive amounts of blood cells clumping along the endothelium (Figure 1(b)). This striking finding was not observed in the other groups.

The aortic sections stained with VVG showed normal histological features of elastic fibres in the Sham, OVX+MCLD, and OVX+MCHD (Figures 1(f), 1(i), and 1(j)) where the internal elastic laminae (IEL) appeared continuous, thick, and wavy. However, there was thinning and disruption of IEL in the TM of OVX and OVX+ST groups (Figures 1(g) and 1(h)).

**Figure 2:**
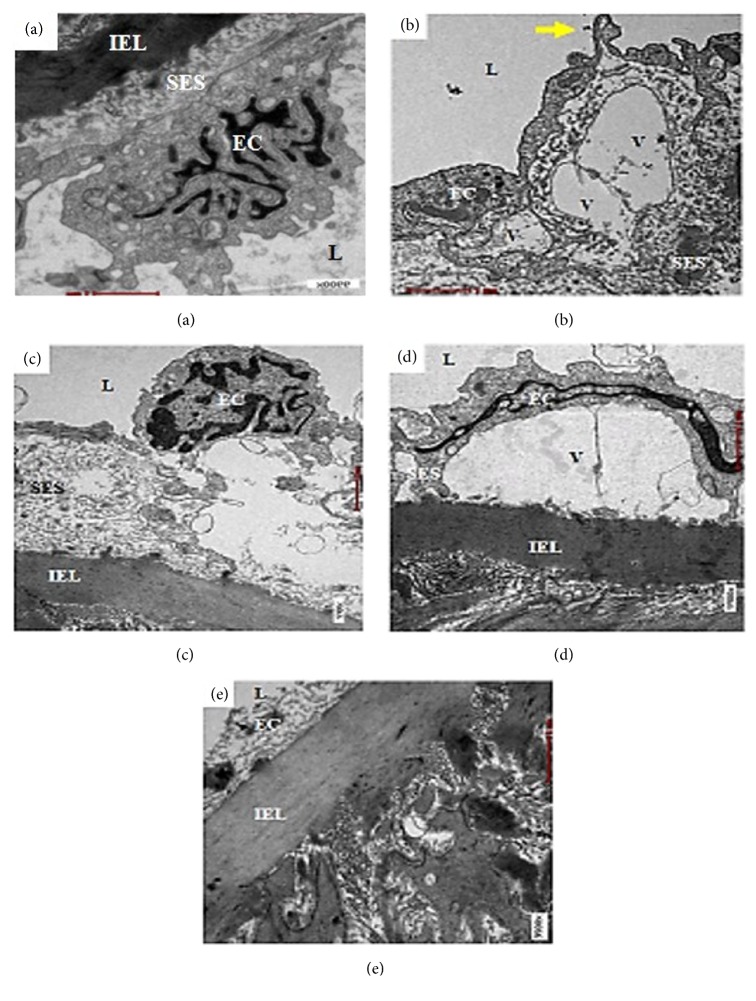
Electron micrograph showing endothelium of the thoracic aorta of (a) Sham, (b) OVX, (c) OVX+ST, (d) OVX+MCLD, and (e) OVX+MCHD. Vacuolation (V) of the endothelium with hook-like cytoplasmic process (arrow) and widened subendothelial space (SES) filled with granular substances were noted in (b) OVX. L: lumen, EC: endothelial cell, IEL: internal elastic lamina. EM X9900.

**Figure 3:**
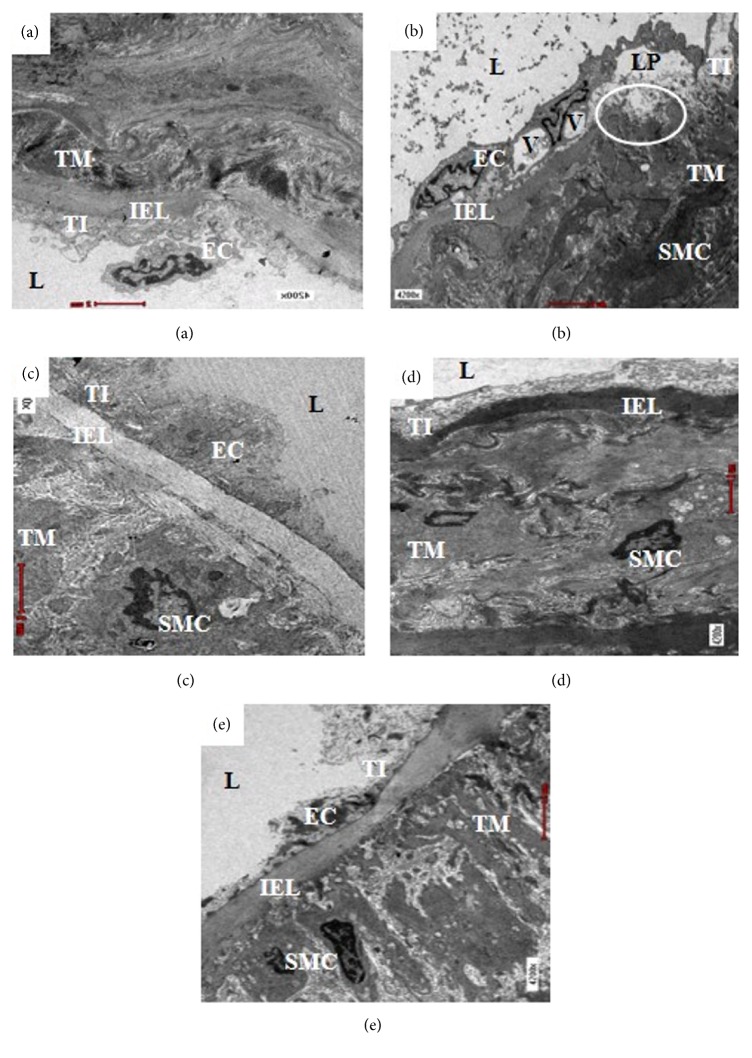
Electron micrograph showing ultrastructure of the thoracic aorta of (a) Sham, (b) OVX, (c) OVX+ST, (d) OVX+MCLD, and (e) OVX+MCHD. Vacuolation (V) and lipid accumulation (LP) were noted in (b) OVX, also disruption of internal elastic lamina (IEL) with migration of smooth muscle cell (SMC) from the tunica media (TM) into tunica intima (TI). The lumen (L) of (b) OVX is filled with numerous granular substances, which was not seen in other groups. EM X4200.

In qualitative electron microscopic observation of the aortic endothelium, normal architecture of the EC was present in the Sham and OVX+MCHD groups (Figures 2(a) and 2(e)). The EC of the OVX and OVX+MCLD groups showed vacuolation which involved large area of the intima (Figures 2(b) and 2(d)). Hook-like cytoplasmic process was seen on the surface of the vacuolated EC in the OVX group (Figure 2(b)). The subendothelial space (SES) appeared widened and filled with massive amount of granular substances in the OVX and OVX+ST groups as demonstrated by the distantly placed IEL (Figures 2(b) and 2(c)). Normal IEL which appeared as a thick and continuous layer separating the TI and TM was observed in the Sham, OVX+ST, OVX+MCLD, and OVX+MCHD groups (Figures 3(a), 3(c), 3(d), and 3(e)). However, the IEL in the untreated OVX group appeared disrupted and discontinuous with the TM contents streamed upward into the TI layer. Vacuolation (V) of the EC was also noted in the TI. Fragmented smooth muscle cells (SMC) were observed in the TM. In addition, massive number of granular substances were seen circulating in the lumen of the vessel giving it a chaotic appearance (Figure 3(b)).

## 4. Discussion

Recent advances in biomolecular sciences have exposed and illuminated the role of inflammation as the main aetiology of atherosclerosis. The understanding of atherosclerosis as a lipid storage disorder is outdated and lacks relevance [26]. Ironically, the current treatment protocol is not targeted on treating and preventing the inflammation. The use of statins as a primary prevention of cardiovascular disease (CVD) is poorly justified and requires more histological investigation and confirmation (Okuyama et al., 2015).

The basis of using ovariectomized rats in this study was to simulate postmenopausal oestrogen deficiency in human. Additionally, we used TPO diet in order to reflect the actual human diet in reality where most of our food are prepared by using reheated oil. This TPO-induced atherosclerosis in postmenopausal rats is according to the methodology of Adam et al., 2009, with slight modification. The reason of choosing MC leaf extract instead of fruit extract in this study is because MC leaf is a highly multifunctional medicinal food used in Traditional Malay Medicine to treat and prevent chronic diseases [22]. The nutritional composition of MC leaf includes water 89.1 g, protein 3.9 g, fat 0.6 g, carbohydrate 2.2 g, fibres 3.0 g, calcium 114 mg, phosphorus 14 mg, ion 2.8 g, sodium 18 mg, potassium 234 mg, *β*-carotene 3619 ug, vitamin B1 1.5 mg, vitamin B2 0.32 mg, niacin 1.0 mg, and ascorbic acid 115 mg [27].

After 3 months of TPO feeding, the untreated OVX rats developed hypertension as indicated by the significantly elevated DBP. This finding is in accordance with the previous study done by Ng et al., 2012. Repeated heating of the palm oil at high temperature causes physical and chemical changes in the oil at molecular level such as hydrolysis, oxidation, and polymerization [7]. Lipid oxidation causes deterioration in the chemical composition of the palm oil by oxidizing the fatty acids [28] and producing polar compounds and polymeric products [29, 30]. The presence of oxidation products caused imbalance between vasodilator prostacyclin (PGI_2_) and vasoconstrictor thromboxane A_2_ (TXA_2_) [31] leading to the development of hypertension [32]. In this study, we found that supplementation with high dose MC significantly reduced the SBP. This finding is in agreement with the previous studies where treatments with MC fruit juice and root extract were reported to have antihypertensive effect [33–35]. The mechanism of antihypertensive effect of MC was through the inhibition of angiotensin converting enzyme (ACE) [33] and by its vasodilatory property [36]. Therefore we postulated that the bioactive compounds which have antihypertensive activity are present not only in the fruit and root of MC but also in the leaf.

Chronic consumption of TPO in postmenopausal rats was reported to cause atherogenic pattern in the lipid profile by increasing the TC, TG, and LDL and decreasing the HDL [37]. In contrast, we observed no significant elevation in the serum TC, TG, and LDL in all groups. This discrepancy occurred because 2% cholesterol was not added in our test diet. Serum TG was not elevated in this study probably because the liver acts as a buffer that rapidly stores the excess circulating lipids [38]. However, low HDL level was observed in the untreated OVX group in accordance with the previous findings [37]. This could be explained by the conversion of the circulating free fatty acids from the TPO diet to very low-density lipoprotein (VLDL) which suppressed the HDL synthesis via cholesteryl ester transfer protein [39]. Significantly higher TC/HDL seen in the OVX and OVX+MCLD indicates higher risk of developing CVD in both groups [10]. Low dose MC could not decrease the TC/HDL probably because the low dose was insufficient to promote therapeutic effects. Transient increase in LDL was reported after TPO feeding [37]. The LDL particle is susceptible to oxidative structural modification to form oxidized LDL (ox-LDL) which activates vascular inflammation. Increased LDL in the circulation leads to an increase in the number of LDL entering the intima [40]. According to oxidation hypothesis of atherosclerosis, LDL is isolated and retained in the intima through proteoglycan binding [41]. Once trapped inside the intima, it undergoes oxidation to produce lipid hydroperoxides, lysophospholipids, and carbonyl compounds [42]. These modified lipids are highly inflammatory molecules because they can induce the release of adhesion molecules, chemokines, proinflammatory cytokines, and other mediators of inflammation in macrophage and vascular wall. They can also undergo further modification in the vascular wall making them antigenic by invoking T cell responses [43]. Relevant to this hypothesis, we found no significant elevation in the serum LDL in all groups which suggest that the LDL has undergone oxidation and trapped inside the vascular wall. Similar results were found in the study done by Adam et al., 2008 [44]. Treatment with high dose MC significantly reduced the serum TC even in the absence of dyslipidemia. This result is in accordance with the previous study done by Mandukhail et al., 2010, who reported that MC leaf extract contains flavones [36] which are able to inhibit lipid biosynthesis [45]. However, the mechanism of TC lowering effect observed in our study was due to lipids elimination through the stools. It means the toxic TPO was effectively eliminated from the body instead of being absorbed into the circulation, thus preventing vascular inflammation and plaque formation at the earliest stage. We observed that the groups treated with MC have steatorrhea compared to the normal Sham group, whereas the untreated group was observed to have constipation.

Hypertension and atherogenic lipid profile are classical risk factors for the genesis of atherosclerosis in this study [26]. Histological evaluation of the aortae of the untreated OVX rats fed with TPO diet showed evident atherosclerotic changes. Accumulation of numerous blood cells in the luminal aspect of the aortic wall indicates the presence of active and ongoing inflammation. The blood cells were seen adhering and clumping around the vessel wall. Theoretically, vascular inflammation can be observed in various stages: stasis of blood due to increase concentration of cells in the blood, leukocytes margination and adhesion along the endothelium, and transmigration across the cell via phagocytosis [46]. All these findings were noted to be present in the OVX group except for the phagocytosis. There was no significant thickening of the TI in all groups. This result was not in accordance with those reported by Xian et al., 2012, because the duration of TPO feeding was shorter in our study. The group treated with statin showed endothelial thickening histologically but further histomorphometric measurement showed no significant increase in the TI thickness. Total disruption of the internal elastic laminae (IEL) observed under VVG staining confirmed the presence of aortic pathology in the untreated OVX group. Thickening of the TM was also noted in the OVX group which was further confirmed by histomorphometric results. Significant increase in TM and IMT indicates the presence of subclinical atherosclerosis [47]. These findings are in contrast with the previous study by Xian et al., 2012. This discrepancy occurred because male rats were used in that study. Another vital finding in this study was the presence of perivascular adipose tissue (PVAT) infiltrating the TA in the untreated OVX and the statin treated group. We also noted that whenever there is PVAT deposition in the adventitia, inflammatory cells are also present. The reason behind this observation is because adipose tissue is a site of inflammation and cytokines production. When PVAT gets inflamed, atherosclerosis is accelerated [48]. According to the “Outside In Theory” of atherosclerosis, inflammation begins in the tunica adventitia (TA) instead of tunica intima (TI) and spreads inwards into the vasculature [49]. In this study, we did observe the lack of boundary between the TA and the surrounding PVAT which was in accordance with the description of Chaldakov et al., 2007 [50]. Neovascularization from the vasa vasorum into the PVAT provides a site of entry for leukocytes into the atherosclerotic lesion which in long term may cause intraplaque haemorrhage and thrombin production. Based on this findings, we do not recommend the use of statin in the absence of dyslipidemia or as a primary prevention of CVD as it causes PVAT accumulation in the aorta. This result is in agreement with expert review by Okuyama et al., 2015, who stated that statin can cause atherosclerosis. Ultrastructural evaluation of the aortae of the untreated OVX rats fed with TPO diet revealed early intimal lesions such as endothelial vacuolation, the presence of hook-like cytoplasmic process, and widening of the subendothelial space filled with massive amount of granular substances, as described by Still & O'Neal, 1962. Apart from the evident endothelial damage, ruptured IEL with SMC proliferation and fragmentation were also observed in the OVX group. The groups treated with statin and low dose MC also showed endothelial vacuolation probably because the treatment dose was ineffective. Treatment with MC leaf extract maintained the normal histology and ultrastructure of the aorta comparable to that of the Sham group. The mechanism of action of MC leaf is due to the presence of flavanoids such as rutin, kaempferol, and quercetin which showed inhibitory effects against proinflammatory cytokines (TNF-*α*, IL-1B, and NO) [19]. Β-carotene in the MC leaf also acts as a potent antioxidative agent [14]. The presence of these phytoactive substances may explain the potent anti-inflammatory and antiatherosclerotic effects of MC observed in our study.

## 5. Conclusion

Chronic consumption of TPO diet in postmenopausal subjects appeared to induce hypertension, atherogenic lipid profile, and recognized histological and ultrastuctural features of atherosclerosis. Treatment with MC leaf was shown to maintain the normal aortic histology, histomorphometry, and ultrastructure. In conclusion, we reported the protective effects of MC leaf against atherosclerosis and validated its therapeutic uses in Traditional Malay Medicine.

## Figures and Tables

**Table 1:** Evaluation of blood pressure and serum lipid profile following MC treatment.

Variable	Sham	OVX	OVX+ST	OVX+MCLD	OVX+MCHD
Blood pressure					
SBP (mmHg)	108.71 ± 2.87	120.0 ± 1.62	116.25 ± 4.0	113.0 ± 2.92	106.13 ± 3.54^#^
DBP (mmHg)	72.43 ± 2.71	90.43 ± 2.33^*∗*^	80.38 ± 3.17	79.50 ± 2.67	74.88 ± 2.84
MAP (mmHg)	80.29 ± 3.84	100.57 ± 1.76^*∗*^	91.25 ± 3.89	79.5 ± 3.88^#^	84.25 ± 2.74^#^
Serum lipid profile					
TC (mmol/L)	0.78 ± 0.06	0.98 ± 0.05	0.76 ± 0.10	0.72 ± 0.06	0.49 ± 0.10^#^
HDL (mmol/L)	0.67 ± 0.05	0.43 ± 0.04^*∗*^	0.55 ± 0.06	0.49 ± 0.06	0.63 ± 0.03
LDL (mmol/L)	0.86 ± 0.10	0.67 ± 0.07	0.95 ± 0.13	1.09 ± 0.17	1.08 ± 0.14
TG (mmol/L)	1.23 ± 0.17	1.47 ± 0.18	1.05 ± 0.19	1.04 ± 0.22	1.41 ± 0.17
TC/HDL (mmol/L)	3.11 ± 0.12	4.10 ± 0.16^*∗*^	3.84 ± 0.16	4.25 ± 0.40^*∗*^	3.88 ± 0.11

Values are mean ± SEM, n=7-8, *∗* significant difference from Sham, # significant difference from OVX (P<0.05).

**Table 2:** Evaluation of aortic histomorphometry following MC treatment.

Variable	Sham	OVX	OVX+ST	OVX+MCLD	OVX+MCHD
TI (*μ*m)	8.19 ± 0.69	8.90 ± 1.38	9.95 ± 1.22	9.60 ± 1.05	8.43 ± 0.78
TM (*μ*m)	150.7 ± 2.81	168.24 ± 5.79^*∗*^	160.52 ± 3.22	159.6 ± 2.64	153.06 ± 3.28^#^
IMT (*μ*m)	158.89 ± 3.2	178.56 ± 6.56^*∗*^	170.47 ± 3.87	169.2 ± 3.22	161.48 ± 3.9^#^

Values are mean ± SEM, n=7-8, *∗* significant difference from Sham, # significant difference from OVX (P<0.05).

## Data Availability

The data supporting the findings in this study are available from the corresponding author upon request.
